# Permanent LEft ventricular septAl Pacing vs. right ventricular pacing in patients with atrioventricular conduction disorders—a randomized trial: rationale and design of the LEAP trial

**DOI:** 10.1093/ehjopen/oeag108

**Published:** 2026-08-11

**Authors:** Jesse H J Rijks, Johan van Koll, Haran Burri, Karol Curila, Vincent F van Dijk, Arnaud Hauer, Jurren M van Opstal, Jan de Pooter, Leonard M Rademakers, Stefan A J Timmer, Wouter Wieringa, Brigitte A B Essers, Sander M J van Kuijk, Hans-Peter Brunner-La Rocca, Kevin Vernooy, Justin Luermans

**Affiliations:** Department of Cardiology, Cardiovascular Research Institute Maastricht (CARIM), Maastricht University Medical Centre (MUMC+), P. Debyelaan 25, PO Box 5800, 6202 AZ Maastricht, The Netherlands; Department of Cardiology, Cardiovascular Research Institute Maastricht (CARIM), Maastricht University Medical Centre (MUMC+), P. Debyelaan 25, PO Box 5800, 6202 AZ Maastricht, The Netherlands; Department of Cardiology, University Hospital of Geneva, Rue Gabrielle Perret Gentil 4, 1205 Geneva, Switzerland; Cardiocenter, University Hospital Kralovske Vinohrady, 3rd Faculty of Medicine, Charles University in Prague, Šrobárova 1150/50, 100 00 Prague, Czech Republic; Department of Cardiology, St. Antonius Hospital, Koekoekslaan 1, 3435 CM Nieuwegein, The Netherlands; Department of Cardiology, Reinier de Graaf Gasthuis, Reinier de Graafweg 5, 2625 AD Delft, The Netherlands; Department of Cardiology, Medical Spectrum Twente, Koningstraat 1, 7512 KZ Enschede, The Netherlands; Heart Centre, Ghent University Hospital, Corneel Heymanslaan 10, 9000 Ghent, Belgium; Department of Cardiology, Catharina Hospital, Michelangelolaan 2, 5623 EJ Eindhoven, The Netherlands; Department of Cardiology, Noordwest Ziekenhuisgroep, Wilhelminalaan 12, 1815 JD Alkmaar, The Netherlands; Department of Cardiology, Jeroen Bosch Hospital, Henri Dunantstraat 1, 5223 HZ Den Bosch, The Netherlands; Department of Clinical Epidemiology and Medical Technology Assesment (KEMTA), Maastricht University Medical Center (MUMC+), P. Debyelaan 25, PO Box 5800, 6202 AZ Maastricht, The Netherlands; Department of Clinical Epidemiology and Medical Technology Assesment (KEMTA), Maastricht University Medical Center (MUMC+), P. Debyelaan 25, PO Box 5800, 6202 AZ Maastricht, The Netherlands; Department of Cardiology, Cardiovascular Research Institute Maastricht (CARIM), Maastricht University Medical Centre (MUMC+), P. Debyelaan 25, PO Box 5800, 6202 AZ Maastricht, The Netherlands; Department of Cardiology, Cardiovascular Research Institute Maastricht (CARIM), Maastricht University Medical Centre (MUMC+), P. Debyelaan 25, PO Box 5800, 6202 AZ Maastricht, The Netherlands; Department of Cardiology, Cardiovascular Research Institute Maastricht (CARIM), Maastricht University Medical Centre (MUMC+), P. Debyelaan 25, PO Box 5800, 6202 AZ Maastricht, The Netherlands

**Keywords:** Left bundle branch area pacing, Left ventricular septal pacing, Left bundle branch pacing, Bradycardia pacing, Physiologic pacing

## Abstract

Permanent cardiac pacing is the only available therapy in patients with atrioventricular (AV) conduction disorders and can be lifesaving. However, conventional right ventricular pacing (RVP) causes dyssynchronous electrical and mechanical activation of the ventricles, possibly resulting in pacing-induced cardiomyopathy and heart failure. Left bundle branch area pacing (LBBAP) might overcome these adverse effects of RVP, but evidence to support this assumption is lacking. The LEAP trial is a multi-centre investigator-initiated, prospective, randomized controlled, open-label, blinded endpoint evaluation (PROBE) study, designed to test the hypothesis that in patients with an indication for pacemaker implantation due to AV conduction disorders with an expected ventricular pacing percentage ≥20%. LBBAP is superior to RVP in preventing the composite primary endpoint of (1) all-cause mortality, (2) hospitalization for heart failure, and (3) a more than 10% point decrease in left ventricular ejection fraction (LVEF) leading to an LVEF below 50%. The LEAP trial randomizes 470 patients 1:1 to the index LBBAP or RVP control arm. The primary endpoint will be determined at 1-year follow-up. This article describes the design and analytic plan for the trial.

## Introduction and rationale

Permanent cardiac pacing is the only available therapy in patients with bradycardia because of atrioventricular (AV) conduction block. For decades, the standard approach for permanent cardiac pacing has been right ventricular pacing (RVP). However, RVP results in a non-physiologic, dyssynchronous ventricular activation.^1–4^ This dyssynchronous activation may lead to a pacing-induced cardiomyopathy in up to 36%^5^ of patients, possibly leading to heart failure in the long run.^2,5–8^

Left bundle branch area pacing (LBBAP) has been proposed as a more physiologic pacing approach than conventional RVP. Several LBBAP entities have been distinguished, consisting of left ventricular septal pacing (LVSP), left bundle branch pacing (LBBP), and left bundle fascicular pacing (LBFP).^9^ Although in theory capture of the left-sided Purkinje system (LBBP and LBFP) provides the most physiological left ventricular (LV) activation, a substantial amount of patients end up being treated with LVSP.^10^ In animal studies, short- and long-term LVSP has been shown to maintain regional cardiac mechanics, contractility, relaxation, and efficiency, whereas RVP diminished these variables.^11,12^ A large European multi-centre registry demonstrated the feasibility and safety of LBBAP as a primary pacing technique in bradyarrhythmias.^13^

Although LBBAP provides a novel, theoretically more physiological and synchronous pacing approach than traditional RVP, and non-randomized and small randomized studies suggest the potential for clinical benefits from this pacing approach,^6,13–18^ to our knowledge, no large randomized clinical trial (RCT) has yet been performed. We describe here the design of the LEAP trial, a multi-centre randomized controlled trial comparing the effects of LBBAP with RVP in patients with AV block.

### Study design

The LEAP trial is a multi-centre investigator-initiated, prospective, randomized controlled, open-label, blinded endpoint evaluation (PROBE) trial, which is designed to test the hypothesis that LBBAP is superior to the current standard of care with RVP.

**Figure 1:**
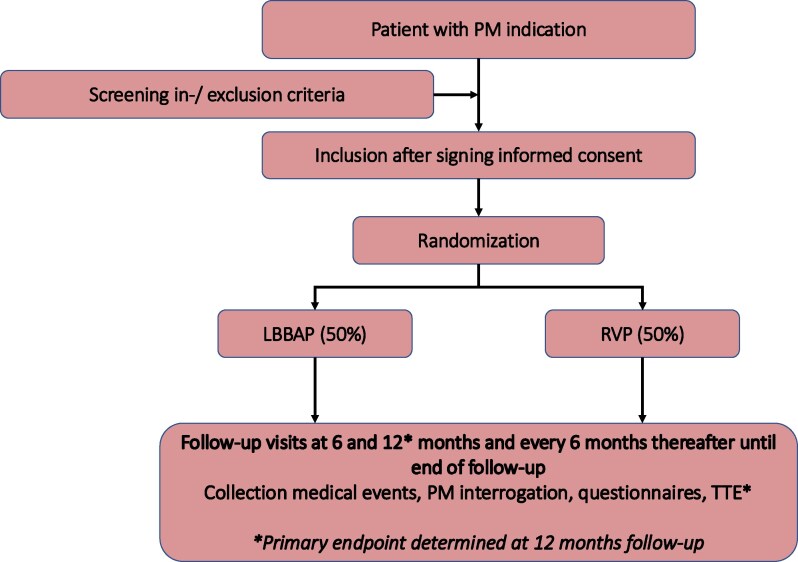
Trial design. PM, pacemaker; LBBAP, left bundle branch area pacing; RVP, right ventricular pacing; TTE, transthoracic echocardiogram. *TTE was only performed at baseline and 12 months follow-up.

Patients who meet the inclusion and exclusion criteria (*Table 1*) are randomized in a 1:1 ratio to LBBAP or RVP after signing informed consent. The study was approved by the medical ethical committee of the Maastricht University Medical Center, the Netherlands (METC 20–029). The LEAP trial is registered at www.clinicaltrials.gov (NCT 04595487). The Trial design is provided in *Figure 1*.

**Table 1:** Inclusion and exclusion criteria of the LEAP study

Inclusion criteria	Exclusion criteria
Age ≥ 18 years Life expectancy with good functional status of ≥ 1 year Class I or IIa PM indication due to AV conduction disorder Acquired third or second degree AVB Atrial arrhythmia with slow ventricular conduction Expected VP percentage ≥ 20% LVEF ≥40% on TTE ≤ 3 months before PM implantation Signed and dated ICF	HF NYHA classes III and IV Class I indication for CRT Class I indication for ICD Previous implanted CIED (except for ILR) Atrial arrhythmia with planned AV junction ablation ACS or unstable angina PCI or CABG ≤30 days before enrollment VHD with indication for valve repair or replacement HCM with IVS thickness > 2 cm
	Renal insufficiency requiring HD Active infectious disease or malignancy Pregnancy

ACS, acute coronary syndrome; AV, atrioventricular; AVB, atrioventricular block; CABG, coronary artery bypass graft; Class I, pacemaker implantation is indicated^19^; CIED, cardiac implantable electronic device; Class IIa, pacemaker implantation should be considered^19^; CRT, cardiac resynchronization therapy; HCM, hypertrophic cardiomyopathy; HD, haemodialysis; HF, heart failure; ICD, implantable cardioverter defibrillator; ICF, informed consent form; ILR, implantable loop recorder; IVS, interventricular septum; LVEF, left ventricular ejection fraction; NYHA, New York Heart Association classification; PCI, percutaneous coronary intervention; PM, pacemaker; VHD, valvular heart disease; VP, ventricular pacing.

### Patient population

The trial is performed in adult patients with a permanent pacing indication because of AV block (class I or IIa according to the 2021 ESC guidelines^19^) with an expected ventricular pacing percentage of ≥ 20% and a left ventricular ejection fraction (LVEF) ≥ 40%. Inclusion and exclusion criteria are provided in *Table 1*.

### Randomization, blinding, and treatment allocation

Patients are randomized in a 1:1 ratio via an interactive response technology system by block randomization. Random permuted block sizes will be used to prevent prediction of treatment allocation before randomization. Randomization will be stratified by sex and percentage of LVEF (i.e. LVEF <50% and LVEF ≥50%) to ensure an even distribution of these characteristics between the two treatment groups. Given the nature of the treatment arms, the participating patients and treatment team are not blinded. Adjudication of the endpoints is performed by a blinded committee.

## Study procedures

### Intervention group

The intervention group is treated with LBBAP. LBBAP consists of the implantation of a pacemaker with the ventricular lead delivered through the interventricular septum to the LV septum, targeting capture of the LBB. However, if it is not possible to capture the LBB, LVSP is considered adequate as well.^9^ The feasibility and safety of this implantation method have been described previously.^13,14,20^

In case of unsuccessful lead positioning in the LBBAP region, the lead may be placed at the His bundle region or in the right ventricle according to the physician’s discretion.

### Control group

The control group is treated with RVP (current standard of care). The ventricular pacing lead is placed in the RV according to the routine of the implanting physician.

### Diagnostic work-up and follow-up

The diagnostic work-up prior to pacemaker implantation includes recording of medical history and medication use, physical examination, 12-lead ECG, and a baseline transthoracic echocardiogram (TTE) no longer than 3 months before pacemaker implantation. Patients will be followed until the last included patient reaches the 1-year follow-up mark.

Pacemaker check-ups following the intervention are performed at 1 month (±2 weeks), 6 months (±2 months), 12 months (± 2 months), and thereafter every 6 months. VVI-paced 12-lead ECG recordings, electrical lead measurements (including sense, threshold, and impedance for all the implanted leads), percentage of ventricular pacing, and atrial high rate episodes (AHRE)/atrial fibrillation (AF) episodes lasting more than 24 h are collected during these checks.

All patients visit the physicians’ outpatient clinic at 6 (±2 months) and 12 months (±2 months) after pacemaker implantation and every 6 months thereafter (follow-up at 18 and 30 months may be performed as a remote visit, i.e. phone consultation) until a maximal follow-up of 36 months. The occurrence of adverse medical events, death, hospitalization (for heart failure), AF, pacemaker-related complications, and New York Heart Association classification (NYHA) class will be noted during these visits. An additional check of adverse events and endpoints will be performed after the last included patient reaches the 1-year follow-up mark.

In addition to the baseline TTE prior to pacemaker implantation, a follow-up TTE is performed at 1 year after implantation. LVEF is determined according to the modified Simpson’s method. Moreover, LV dimensions, the presence of valvular abnormalities, and diastolic function are graded. Diastolic function will be assessed by determining the following echocardiographic parameters at baseline and 1-year follow-up: E-wave, A-wave, E/A ratio, septal and lateral e’, E/e’, 2D-strain of the left ventricle and atrium, pressure gradient across the tricuspid valve, and volume of the left atrium. Diastolic function will be graded according to the recommendations by Nagueh et al.^21^ Diastolic dysfunction assessment will be limited by the presenting rhythm at baseline echocardiography.

At baseline and every 6 months thereafter until the end of the study, the EQ-5D-5L questionnaire is used to assess health-related quality of life (QOL), and the iMTA Productivity Cost and iMTA Medical Consumption Questionnaire are used for the calculation of costs outside of the hospital.

## Endpoints

### Primary endpoint

The primary endpoint is the composite of all-cause mortality, hospitalization for heart failure, and a more than 10%-point decrease in LVEF leading to an LVEF below 50%, which as a binary combined endpoint will be determined at 1-year follow-up.

Heart failure hospitalization is defined as:

1. Hospitalization or unplanned hospital visits for heart failure requiring intravenous diuretics and/or (adjustment) of other medical heart failure therapy.
2. Or hospitalization for upgrade to cardiac resynchronization therapy (CRT) (biventricular pacing) for progression of heart failure with worsening NYHA class and/or (increased) need for diuretic therapy or other medical heart failure therapy.
3. Or hospitalization for upgrade to CRT for developing a class I indication for CRT.
4. Or hospitalization or unplanned hospital visit for heart failure triggered by an episode of atrial fibrillation with uncontrolled heart rate requiring intravenous diuretics or adjustment of rate control therapy.

### Secondary endpoints

Secondary endpoints are:

- Time to first occurrence of all-cause mortality or hospitalization for heart failure.
- Time to first occurrence of all-cause mortality.
- Time to first occurrence of hospitalization for heart failure.
- Time to first occurrence of AF *de novo* and AF burden at every follow-up visit.
- The echocardiographic change in LVEF at 1 year as compared to baseline.
- The echocardiographic changes in diastolic (dys-)function at 1 year as compared to baseline.
- The occurrence of pacemaker-related complications.
- QOL assessed by the EQ-5D-5L questionnaire.
- Cost-effectiveness analyses and budget impact analysis (BIA).

The secondary endpoints (other than echocardiographic endpoints) will be determined at the end of the follow-up period when the last included patient has reached 1-year follow-up. The individual follow-up time for patients at this time point will vary, with a minimum of 1 year and a maximum of 36 months.

### Adjudication of endpoints

All clinical endpoints as described above are adjudicated by an independent, blinded endpoint adjudication committee (EAC). The EAC consists of three experienced cardiologists. Two of them adjudicate all clinical endpoints. In case of disagreement, after discussion with the third committee member, they decide on the adjudication of the endpoint. All endpoints are adjudicated using standardized definitions provided in the EAC charter.

An independent echo core lab, composed of two experienced imaging cardiologists, assesses all baseline and follow-up TTE’s. Based on the baseline and 1-year follow-up LVEF, as assessed by the echo core lab, the echocardiographic part of the primary endpoint is determined. The echo core lab is blinded for the study number and date of the examination. Due to the presence of pacing leads, the core lab cannot be completely blinded to the allocation of the patient.

An ECG core lab, consisting of two experienced LBBAP implanters, assesses all paced baseline and 1-year follow-up ECG’s to determine the presence of RVP, deep septal pacing, or LBBAP according to the previously published definition.^9^ Capture of the left-sided Purkinje system is determined according to the current consensus.^9^

## Analyses

### Sample size calculation

Based on previously reported mortality, heart failure hospitalizations, and significant deterioration of LVEF,^2,6–8,22^ we estimate that the proportion of patients in the RVP group that will develop any of the composites of the primary endpoint before 1-year follow-up will be at least 25%. We expect an event rate of 14% for the LBBAP group. Event rates are based on the effect size of heart failure-related events by his pacing in those with ventricular pacing percentage >20%.^6^ When designing this trial, no data on event rates in LBBAP were available yet. The effect size of LBBAP was estimated to be equal to HBP when designing this trial. To achieve a power of 80% at the 5% level of significance, 203 patients per group, or 406 in total, would be needed. Accounting for around 15% loss to follow-up or patients not receiving the allocated intervention, 470 patients are needed.

After designing the trial, Sharma et al. reported an occurrence of the combined primary clinical endpoint of mortality, heart failure hospitalization, or upgrade to CRT among patients with ventricular pacing burden > 20%, in 26.1% in RVP and 8.4% in LBBAP after a mean follow-up of 1.5 years in a non-randomized trial (HR 0.32; 95% CI 0.19–0.54; *P* 0.001).^15^ This study included a longer follow-up duration but excluded the echocardiographic endpoint. The addition of the echocardiographic endpoint in the LEAP trial will generate more endpoints in a shorter follow-up duration. Therefore, the study by Sharma et al. is substantiating the sample size calculation.

### Statistical analyses

Analyses will be executed on an intention-to-treat population. This population includes all patients who are randomized, regardless of the treatment they received. For example, all patients randomized to LBBAP and not receiving LBBAP (i.e. RVP or HBP) will be analysed according to their randomization group (thus LBBAP). The same counts for all patients randomized to RVP and not receiving RVP.

Given the possibilities of crossovers, as a secondary analysis, an exploratory per-protocol analysis will be performed to check for consistency and explore potential efficacy. The per-protocol analysis will include all patients randomized to LBBAP who received LBBAP and all patients randomized to RVP who received RVP.

The ECG corelab will assess all paced baseline and 1-year follow-up ECGs to determine the pacing type the patients are treated with. Descriptive statistics (i.e. count (%)) will be used to display the actual pacing types (i.e. RVP, LBBAP, or HBP) in both treatment groups.

Categorical baseline characteristics will be presented as count (percentage). Continuous characteristics will be presented as mean (± standard deviation) or median (interquartile range) as appropriate.

All primary and secondary study endpoints will be described in detail as count and percentage (binary endpoints), using Kaplan–Meier curves (time-to-first occurrence of an endpoint), Cox regression analysis, and as mean and standard deviation (continuous endpoint, i.e. LVEF), depending on the type of outcome. All null-hypothesis testing will be performed using a level of significance of α = 0.05.

### Statistical analyses of the primary endpoint

The analysis of the primary composite endpoint at 1 year after treatment will be performed using logistic regression analysis, adjusted for sex, and LVEF category, as these were stratification variables for randomization. Missing echocardiographic data will be imputed using multiple imputation with fully conditional specification. The amount of imputations will be set to the percentage of incomplete records. Predictive mean matching will be used to draw imputations, retaining the original distributional characteristics. A complete case analysis will be used as a sensitivity analysis.

### Statistical analyses of the secondary endpoint

For the secondary endpoints, the treatment groups will be compared using Cox proportional hazards regression, again adjusted for the stratification variables used in the randomization procedure. The secondary endpoints are analysed without corrections for multiplicity. Hazard ratios will be presented with associated 95% confidence interval. The proportional hazards assumption will be tested as the association between the scaled Schoenfeld residuals and follow-up time. Change in LVEF will be compared between groups using multivariable linear regression analysis. We will use multivariable generalized linear regression analysis with a link-function dependent on the specific distribution to assess between-group differences in diastolic function.

### Cost-effectiveness analysis

A trial-based economical evaluation from a societal perspective will be performed in accordance with the Dutch guidelines for economic evaluations in healthcare.^23^

### Cost analysis

Resource use will be measured from a societal perspective using data from case record forms and the medical consumption (MCQ) and productivity loss (PCQ) questionnaires. Cost categories that will be included are (1) healthcare costs related to the costs of the pacing method and hospital admission; (2) lost productivity costs (absenteeism from paid and unpaid work); and 3) patient costs (informal care and other care services paid for by patients themselves). Valuation of resource use will be done according to the Dutch costing manual or based on calculations that follow the recommendations of this manual.^24^ Medication use will be valued using prices of the Pharmacotherapeutic Compass. For the valuation of absenteeism from paid work, the friction cost approach will be used. For costs, a discount rate of 4% will be applied, following standard practice in the Netherlands.^23^

### Patient outcome analysis

Assessment of QOL is based on the EQ-5D-5L questionnaire at baseline, 6, 12 months follow-up, and every 6 months thereafter until follow-up ends and follows recommendations of the standardized Dutch quality of life measurement manual.^25^ Quality-adjusted life years are calculated from the area under the curve. It is calculated as follows: the value given to a health state multiplied by the time period spent in that state. A discount rate of 1.5% will be applied for the patient outcome, following standardized practice in The Netherlands.

### Data analysis cost-effectiveness

Incremental cost-effectiveness ratio (ICER) is calculated by dividing the difference in costs by the difference in effectiveness between the treatment groups. Differences between the two groups will be analysed with bias-corrected bootstrap analysis. Bootstrap analysis will also be used to quantify uncertainty surrounding ICER. Results of this analysis will be presented in cost-effectiveness planes and acceptability curves. Probabilistic sensitivity analysis will be performed to estimate the impact of both clinical and cost parameters on the ICER.

### Budget impact analysis

BIA will be performed from a societal, health care provider, and health care insurer perspective. The eligible population will be estimated based on national health care data.

Costs of the intervention and heart failure costs will be included. Indirect costs will not be included. The time horizon will be 3 years. The expected uptake rate will be estimated based on a panel of experts (cardiologists, implementation specialists and patient representatives), and analyses will be performed for this expected uptake rate and several slightly higher and lower uptake rates. Uncertainties and scenarios will be discussed in a panel of experts as well, and different scenarios will be analysed. Recommendations of the ISPOR task force are followed for all BIA calculations.^26^

### Monitoring

The Trial Steering Committee has the responsibility for the execution and scientific reporting of the trial. The steering committee comprises a group of experts in clinical cardiology and an expert clinical epidemiologist.

An independent data safety monitoring board consisting of one clinical epidemiologist and two experts in clinical cardiology with expertise in the management of patients in the scope of the study reviews accumulating safety and compliance data of the study. This committee regularly reviews unblinded data according to a charter and gives recommendations to the Trial Steering Committee.

## Discussion

This article describes the design and analytic plan for the LEAP trial. The LEAP trial is a randomized controlled trial with a superiority design, comparing LBBAP with routine RVP in patients with an ESC class I or class IIa indication for pacemaker implantation due to second- or third-degree AV block with anticipated ventricular pacing percentage >20%. In total, 470 patients are randomized in a 1:1 ratio to either the index LBBAP or control RVP and compared with respect to the composite primary endpoint of mortality, heart failure hospitalization, or a 10% point decrease in LVEF leading to an LVEF <50% at 1-year follow-up.

RVP has been the gold standard in pacing for AV conduction disorders for decades^27^ and is still recommended in current practice guidelines for permanent cardiac pacing in patients with an LVEF ≥40%.^19^ The RV is an easily accessible and stable site for lead fixation. However, RVP results in a non-physiological, dyssynchronous activation of the heart, which is associated with an increased risk for heart failure, atrial fibrillation, and even cardiovascular death.^1,4^

In the DAVID trial,^4^ RVP was even associated with heart failure and death in patients with reduced LV function. In the MOST trial,^1^ a higher percentage of RVP was associated with increased heart failure hospitalizations in bradycardia patients with normal ventricular function. Khurshid et al. described an incidence of pacing-induced cardiomyopathy in 50/257 (19.5%) patients with frequent (≥20%) RVP, with a decrease in mean LVEF from 62% to 36% over a mean follow-up period of 3.3 years.^2^

Several alternative pacing strategies, including biventricular pacing (BiVP) and HBP, have been proposed to overcome the adverse effects of RVP.^28^ BiVP consists of the simultaneous stimulation of the right ventricle (RVP) and left ventricle by means of an additional LV lead implanted in a coronary vein tributary. Although BiVP theoretically creates a more synchronous activation of the heart than RVP, it requires an extra lead, the implantation is more complex, associated with more complications, and it still results in a non-physiologic ventricular activation composed of two wave fronts.^29^ Moreover, the LV lead and the biventricular pacemaker device are associated with increased costs. Interestingly, the two largest randomized trials that compared biventricular pacing with RVP (BLOCK HF^8^ and BIOPACE^30^) yielded conflicting results. The BLOCK HF trial showed a significant reduction in the composite endpoint of death from any cause, an urgent care visit for heart failure requiring I.V. therapy, or a 15% or more increase in LV end-systolic volume index, in patients receiving BiVP for AV block over RVP. In contrast, the BIOPACE study showed no significant difference in the co-primary endpoint of (i) time to death or first heart failure hospitalization and (ii) survival time between RVP and BiVP.

Permanent His Bundle Pacing (HBP) theoretically is the most physiological alternative approach to ventricular stimulation.^31^ Since the development of specially designed sheaths and leads for delivering permanent HBP, there have been numerous publications demonstrating its applicability and feasibility.^32^ However, HBP remains challenging with an implantation success rate of only 80–85%, even by experienced operators.^33^ Furthermore, HBP is associated with increased pacing thresholds and sensing issues (ventricular undersensing and atrial oversensing), frequently resulting in a third back-up lead being placed in the RV,^34^ or faster generator depletion.^32^ Due to the aforementioned downsides, until now, HBP could never live up to its physiological potential.

Most recently, LBBAP has been introduced as a simpler and more reliable pacing strategy with a higher success rate and satisfactory pacing and sensing parameters when compared to HBP.^15,35^ The electrical and mechanical physiology of LBBAP has been previously described, and several studies have demonstrated the safety and feasibility with commercially available pacing leads,^14,20,36^ along with its learning curve.^37^ Non-randomized studies have shown the potential of LBBAP in reducing the occurrence of pacing-induced cardiomyopathy and the need for upgrade to CRT.^14,15^ The largest registry-based study to date showed a clear long-term survival benefit in patients with preserved or mildly reduced LVEF receiving LBBAP for AV conduction disorders over RVP.^18^ Two small randomized trials demonstrated a smaller reduction in LVEF^16^ and a reduction in pacing-induced cardiomyopathies^17^ in patients receiving LBBAP for AV conduction disorders when compared to RVP. Unlike the Prague CSP trial,^16^ the CSPACE trial also demonstrated a reduction in CRT upgrades in these patients when receiving LBBAP.^17^ However, no difference in heart failure hospitalizations or mortality was found.^16,17^

In the 2021 ‘European Society of Cardiology (ESC) guidelines on cardiac pacing and cardiac resynchronization therapy’ LBBAP is mentioned as a very promising technique, but no recommendations on its use could be made yet because of the lack of evidence from randomized trials.^19^ Recently, the ESC endorsed a clinical consensus statement on conduction system pacing indications.^38^ Although recommendations for the use of LBBAP were made (may be appropriate to do in patients with AV conduction disorders and an LVEF ≥40%), it was not possible to recommend the use of LBBAP over RVP for these patients because of the lack of evidence.^38^

The LEAP trial aims to provide evidence supporting the superiority of LBBAP over RVP in patients with AV block with respect to the combined clinical endpoint of mortality, heart failure hospitalization, and development of pacing-induced cardiomyopathy in a randomized trial. Furthermore, the trial will investigate whether LBBAP is safe, cost-effective, and results in an improved QOL when compared to RVP.

### Limitations

Due to the chosen design, this trial has several limitations. The composite primary endpoint consisting of both clinical outcomes (death and heart failure hospitalization) and echocardiographic outcome (a more than 10% point decrease in LVEF leading to an LVEF below 50%) has some limitations. First, due to limited data on outcomes in LBBAP regarding mortality, heart failure hospitalization, and echocardiographic changes when compared to RVP, it is unclear how much each part of the composite endpoint will contribute to the overall result. Based on previous reports,^16,17^ the echocardiographic part is expected to contribute for ∼50% or more to the primary endpoint. Second, due to the addition of an echocardiographic endpoint, no time-to-event analysis is possible for the primary endpoint. Therefore, secondary endpoints with time-to-event analyses are added to the protocol.

Moreover, the sample size calculation for the study is mostly based on a study on HBP^6^ because effect sizes for LBBAP were largely unknown when designing this trial. Due to the limited available data and no prospective, retrospective, or randomized studies available on the combined endpoint chosen in this trial, the sample size calculation is based on an estimation. This might result in a less accurate sample size calculation.

Nevertheless, choosing a composite endpoint including only clinical outcomes (i.e. death and heart failure hospitalization) would require a much larger sample size and longer follow-up duration. LBBAP is currently applied on a large scale in routine clinical care without evidence from large, randomized trials. Due to the need for randomized data on LBBAP on a shorter term, the current design was chosen.

## Data Availability

The data underlying this study will be shared on reasonable request to the corresponding author.

## Lead author biography

Jesse Rijks is a cardiologist and PhD candidate in the Maastricht University Medical Center+ in Maastricht, the Netherlands. He started his training as an electrophysiology and device fellow in the Maastricht University Medical Center+ in January 2026. His clinical focus is on cardiac device therapy and treatment of arrhythmias. His research focuses on conduction system pacing, especially LBBAP. He is the study coordinator of the LEAP trial, an international randomized trial comparing LBBAP with right ventricular pacing.
